# Forest succession improves the complexity of soil microbial interaction and ecological stochasticity of community assembly: Evidence from *Phoebe bournei*-dominated forests in subtropical regions

**DOI:** 10.3389/fmicb.2022.1021258

**Published:** 2022-11-28

**Authors:** Gongxiu He, Tieshuang Peng, Yi Guo, Shizhi Wen, Li Ji, Zhong Luo

**Affiliations:** School of Forestry, Central South University of Forestry and Technology, Changsha, China

**Keywords:** forest succession, soil microbial community, community assembly, co-occurrence network, *Phoebe bournei*

## Abstract

Forest succession is a central ecological topic, due to the importance of the associated dynamic processes for terrestrial ecosystems. However, very little is currently known about the community assembly and interaction of soil microbial communities along forest successional trajectories, particularly regarding the microbial community dynamics in contrasting seasons. To bridge these knowledge gaps, we studied soil bacterial and fungal community compositions, assemblages, and co-occurrence networks in a well-established successional gradient of *Phoebe bournei*-dominated forest, spanning about 65 years of forest development in a subtropical region. Illumina MiSeq sequencing of 16S and ITS genes was employed for the assessment of soil bacterial and fungal community composition and diversity, respectively. The relative abundance and α–diversity of soil bacteria and fungi showed a differential trend over forest succession. The dominant fungal phyla (Basidiomycota and Ascomycota) changed more frequently than the dominant bacterial phyla (Proteobacteria, Acidobacteriota, and Actinobacteriota), indicating that soil fungi have a more sensitive relationship with forest succession compared with bacteria. The soil microbial community variation induced by forest succession was significantly affected by soil total phosphorus, dissolved organic carbon content and pH. Compared to deterministic processes, stochastic processes mainly dominated the community assembly of soil microbial communities. Meanwhile, the relative importance of stochasticity in soil fungal communities increased in the later stages. In Particular, dispersal limitation and drift accounted for a large proportion of bacterial and fungal community assembly, respectively. In addition, the co-occurrence networks of soil microbial communities became more complex as succession proceeds. Soil bacteria and fungi exhibited more competition and cooperation along the forest successional gradient. Collectively, our findings suggest that forest succession improves the complexity of soil microbial interactions and the ecological stochasticity of community assembly in *Phoebe bournei*-dominated forests, providing key insights into the relationship between microbial communities and forest succession.

## Introduction

Forest succession is a critical process in which one forest community evolves into another due to natural and/or anthropogenic forces (Finegan, 1984; Powers and Marín-Spiotta, 2017). The natural succession of secondary forests without human disturbance is an effective way to improve soil conditions and restore degraded environments (Zhang et al., 2016; Zhao et al., 2019). The quantity and quality of aboveground input during forest succession can result in variations in soil microbial diversity and community structures (Smith et al., 2015; Shao et al., 2017). The heterogeneity of the understory over different successional stages can lead to striking differences in soil microbial community characteristics and divergent adaptation strategies (Liu et al., 2020). Thus, disentangling the microbial community composition and diversity in the different stages of forest succession may provide significant insights into the driving successional dynamics and microbe-driven nutrient cycles (Fierer et al., 2010).

Soil bacteria and fungi, as important components of terrestrial ecosystems, are responsible for organic matter decomposition and the regulation of nutrient cycling (Prosser et al., 2007; Harris, 2009; Serna-Chavez et al., 2013; Schimel, 2016). Generally, due to their rapid response and high turnover rates, soil microbes can provide additional information (e.g., ecosystem restoration, nutrient cycling, plant productivity) about forest successional trajectory and early indications of ecosystem restoration (Banning et al., 2011). Mounting evidence has shown the divergent strategies of soil bacterial and fungal communities during forest succession, especially the unpredictable patterns in the varied environmental conditions (Fierer et al., 2007; Zhou et al., 2017). Sun et al. (2017) found that from the early to late stage of forest succession, a shift from copiotrophic to oligotrophic communities possibly occurs, as C and N substrate availabilities decline while nutrient-poor ones (or recalcitrant C components) accumulate. In addition, soil fungi are more closely associated with plant communities, and are capable of utilizing root exudates and litter more effectively than bacteria (Sun et al., 2017; Zhong et al., 2018). Zhou et al. (2017) based on a meta-analysis, have revealed that the soil bacterial and fungal communities dominate in the early and late stages of forest succession, respectively. Specifically, soil bacteria are generally characterized by high growth and turnover rates and are considered as copiotrophs, whereas fungi are inclined toward oligotrophic microbes with slow growth and turnover rates (Fierer et al., 2007; Zhou et al., 2017). Although a number of studies have reported on how various stages of forest succession induce changes in soil microbial communities, very little attention has been paid to the seasonal dynamics in the scenario of forest succession. Temporal heterogeneity (with respect to, e.g., soil nutrients, moisture, and litter input) is an important dimension influencing soil microbial diversity and community composition (Smith et al., 2015). In order to determine the comprehensive mechanism during forest succession (Fierer et al., 2010), an urgent understanding of microbial community characteristics across seasonal and successional dynamics is needed.

Identifying the relative contribution of deterministic (e.g., environmental selection, species interactions, niche differentiation) and stochastic (e.g., random birth, death, and dispersal events) processes that control the assembly of soil microbial communities during forest succession is essential for predicting ecosystem responses to environmental changes (Nemergut et al., 2013). Deterministic processes underpin the roles of biotic and abiotic filtering, which can result in great variation in community composition under divergent environmental conditions, while stochastic processes emphasize the contribution of probabilistic dispersal and ecological drift to community composition patterns (Zhou and Ning, 2017). Previous studies have extensively confirmed that the community assembly of plants during forest succession is influenced by patterns of phylogenetic turnover among coexisting species (Bruelheide et al., 2011; Whitfeld et al., 2012; Purschke et al., 2017). In the terms of the soil microbiota, uncovering the fundamental processes of microbial community assembly is of great significance not only to help predict the succession trajectory of community structure, but also to understand the causes of species composition in their habitats, as well as the restoration of subtropical forests (Graham and Stegen, 2017; Zhou and Ning, 2017). Dini-Andreote et al. (2015) have suggested that, during forest succession, the soil microbial community is initially governed by stochasticity, with a progressive increase in deterministic selection as succession proceeds; more importantly, deterministic processes are affected by the variation in the environment-induced soil organic matter contents. In contrast, in a recent study, Liu et al. (2021) have demonstrated that deterministic processes dominate the composition of the soil microbial community in the early stages, while stochasticity increasingly plays a pivotal role in late stages during secondary succession in subtropical forests. Although accumulating research has reported the simultaneous processes of determinism and stochasticity in microbial community assembly (Chase and Myers, 2011; Ferrenberg et al., 2013; Stegen et al., 2013), it is essential to integrate both processes and decipher their relative importance across different forest successional stages and seasons.

*Phoebe bournei* is a protected and valuable evergreen tree species distributed in Chinese subtropical regions (Wang et al., 2013, 2021). The successful mixed planting of *P. bournei* with *Cunninghamia lanceolata* is a useful afforestation strategy to improve soil degradation and forest productivity (Ding et al., 2022). Due to their high-quality wood properties, economic commodity, and ecological benefit, *P. bournei* trees have suffered from over-harvesting and illegal logging, resulting in population declines. The naturally distributed *P. bournei* forests at Mingyueshan farm comprise a well-documented site representing approximately 65 years of forest succession, providing a unique landscape in which to explore soil microbial community characteristics during forest succession in a subtropical region (Wang et al., 2021). Our previous study has revealed that soil P and N contents become limited as forest succession progresses (Wang et al., 2021). In the present study, we set out to investigate the soil bacterial and fungal diversity, community composition, and community assembly along a forest successional trajectory in the dry and wet seasons in Chinese subtropical regions. We hypothesized that: 1) as a result of the distinct life history of soil bacteria and fungi during long-term forest development (Fierer et al., 2007; Zhou et al., 2017), the forest successional trajectory will have more profound effects on the soil microbial community than season, where soil fungi exhibit a more sensitive association with forest succession compared with bacteria; 2) forest succession and season will simultaneously change the ecological processes of community assembly of both soil bacteria and fungi, where deterministic and stochastic processes have an obvious shift along with the dwelling environment (Dini-Andreote et al., 2015); and 3) based on our previous report regarding increasing soil N and P limitation with forest succession in *P. bournei*-dominated forests (Wang et al., 2021), more complex soil microbial community networks will be found in the late stage of forest succession.

## Materials and methods

### Site description and experimental design

The experiments were conducted at Mingyueshan state-owned forest farm, Anfu County, Jiangxi Province (27°33′14” N, 114°30′20′′ E). The mean annual precipitation is 1,553 mm and the mean annual temperature is 17.7°C. This region has a typical subtropical monsoon climate, with distinct wet and dry seasons. The dry season is from October to February, and the wet season is from March to September. The geomorphology is mainly composed of sandstone, shale, and carbonaceous slate. The soil type in this study area is acrisol.

The sites located at the Mingyueshan farm are dominated by *P. bournei* forests, including *P. bournei* monocultures and natural or semi-natural forests. The *P. bournei* monocultures were established in 2003, and the stand age is 15 years old. The semi-natural *P. bournei*-dominated forests were primarily generated from the monoculture plantations without any anthropogenic management. There are three representing ages of the semi-natural *P. bournei* forests (i.e., 25–35, 35–45, and 45–65 years old), which were identified as being in the early, middle, and late stages of *P. bournei* forest succession, respectively. We selected four typical stages of *P. bournei* forests succession as the subject of this study: a) the initial stage of succession (15 years, S1), in a typical *P. bournei* monoculture plantation with density of 92.9%, with *Photinia vulgaris* and *Elaeocarpus decipiens* as the subdominant species; b) the early stage of forest succession (25–35 years, S2), with density of *P. bournei* of 71.8%, subdominated by *Schima superba* and *Cunninghamia* *lanceolata*; c) the middle stage of forest succession (35–45 years, S3), with density of *P. bournei* of 65.30%, followed by *Quercus glauca* and *Magnolia liliflora*; and d) the latter stage of succession (45–65 years, S4), having density of *P. bournei* of 58.6%, with the forest mainly composed of *Elaeocarpus decipiens*, *Cunninghamia lanceolata*, *Quercus glauca*, and *Magnolia liliflora*. For more detailed information, we refer the readers to the study of Wang et al. (2021).

### Experimental design and soil sampling

In April 2018, the abovementioned four successional stages of the *P. bournei*-dominated forests were chosen for the current study; namely initial (S1), early (S2), middle (S3), and late stages (S4) (monocultures, 15 years old; semi-natural forests, 25–35, 35–45, and 45–65 years old, respectively). For each successional stage, three independent representative plots (25× 25 m) with basically similar site conditions were randomly selected. The distance between stages ranged from greater than 300 m to less than 5 km, and the greatest distance between two plots within a stage was less than 120 m. Collectively, a total of 12 sites (four successional stages × three replicate sites) were included in our study area. In April and October 2018 (wet season and dry season, respectively), eight soil samples were collected within each plot by scraping an “S” shape from the top 10 cm of soil using a stainless steel corer (5 cm diameter) after removing the surface litter. These samples were then mixed as one composite sample for each replicate plot. Each soil sample was immediately passed through a 2 mm sterilized sieve, and the visible plant roots, stones, and debris were removed. Each composite soil sample was divided into two subsamples and placed in sterile bags to be processed and used for the following analyses: 1) stored at 4°C for the measurement of soil physicochemical properties; 2) stored at −80°C for DNA extraction of soil microbes.

### Soil physicochemical properties measurement

After shaking a soil–water (1, 5 w/v) suspension for 30 min, the soil pH was measured using a pH meter (PHS-3C, Leici, China). Soil organic carbon (C) content was measured by UV spectrophotometry based on the hydration heat potassium dichromate-sulfuric acid-colorimetry. Soil total nitrogen (N) was measured using an automatic discontinuous chemical analyzer. The total phosphorus (P) content in soils was determined colorimetrically using a UV spectrophotometer (TU-1901, Puxi Ltd., Beijing, China) after wet digestion with HClO_4_-H_2_SO_4_. The soil dissolved organic carbon (DOC), nitrogen (DON), and phosphorus (DOP) contents were extracted with K_2_SO_4_ solution and were measured according to the method described in Wang et al. (2021). The soil nitrate (NO_3_^−^–N) and ammonium (NH_4_^+^–N) contents were determined using a continuous flow analytical system (AA3, Seal Co., Germany).

### DNA extraction, PCR amplification, and illumina sequencing

Soil microbial genomic DNA was extracted from approximately 1 g (wet weight) of soil using a MoBio PowerSoil DNA Isolation Kit (MoBio Laboratories Inc., Carlsbad, CA, United States) following the manufacturer’s instructions. After the soil sample DNA was extracted, the extracted genomic DNA was detected by 1% agarose gel electrophoresis. The fungal universal ITS1 region was amplified with the primers ITS1-F (5′-CTTGGTCATTTAGAGGAAGTAA-3′) and ITS2 (5′-TGCGTTCTTCATCGATGC-3′) (Zheng et al., 2015). The bacterial universal V3-V4 region of the 16S rRNA gene was amplified with the primers 338F (5′-ACTCCTACGGGAGGCAGCAG-3′) and 806R (5′ -GGACTACHVGGGTWTCTAAT-3′) (Ji et al., 2022a). The PCR volumes were 50 μl, containing 30 ng template DNA, 0.3 μl DNA polymerase (Takara Biotechnology, Dalian, CO., LTD), 2 μl of 10 μM of each primer, 5 μl 10× Pyrobest Buffer, and 4 μl 2.50 mM dNTP. PCR amplification was carried out using the following protocol: 95°C for 5 min, followed by 35 cycles (for fungal ITS1 region) or 28 cycles (for bacterial V3-V4 region) at 95°C for 45 s, annealing at 55°C for 50 s, 72°C for 45 s, and a final extension at 72°C for 10 min.

Three PCR products per sample were pooled, purified, and quantified by real-time PCR. Parallel-tagged sequencing was performed on an Illumina MiSeq platform according to standard protocols. Specifically, split reads were merged using FLASH V1.2.11 (Magoč and Salzberg, 2011; Bolger et al., 2014) and sorted into each sample according to the unique barcodes using the QIIME (V1.7.0) software (Wang et al., 2007). The raw data were first screened, and sequences were removed by considering whether their quality scores <20 contained ambiguous bases or did not exactly match the primer sequences and barcode tags. Raw tags with less than 200 bp were removed using the Mothur (V1.37.0) software (Schloss et al., 2009). Chimeras were removed with USEARCH (V8.1.1861), according to the Gold and UNITE reference databases. The taxonomic assignment of 16S rRNA and ITS sequences was determined based on the bacterial SILVA (v138) reference database and fungal UNITE (v7.2) reference database using the RDP Classifier. The high-quality sequences were clustered into operational taxonomic units (OTUs) at a threshold of 97% similarity using the UPARSE pipeline (Ji et al., 2022a). Singletons that occurred only once in the entire dataset were removed from subsequent analyses, in order to reduce overprediction of rare OTUs. The representative OTU sequences were aligned and annotated using the Ribosomal Database Project (RDP) classifier (V14). Both soil bacterial and fungal datasets were rarefied with smallest values before data analysis. Alpha diversities, such as the observed species and Shannon diversity index were analyzed using QIIME (V1.7.0) (Ji et al., 2022a). The raw sequences data was deposited in National Center for Biotechnology Information (NCBI) with Sequence Read Archive (SRA) database under BioProject ID: PRJNA870323 (bacteria) and PRJNA870328 (fungi).

### Statistical analysis

All data were tested for normality (Kolmogorov–Smirnov test) before statistical analysis. Two-way analysis of variance (ANOVA) was used to explore the effects of renewal stages and seasons on soil microbial diversity and composition, using the SPSS software (IBM SPSS Statistics, Chicago, IL, United States). Tukey’s honestly significant difference test was conducted to test differences among different successional stages at *p* < 0.05, whereas a *T*-test was performed to assess the significance of differences between contrasting seasons. Using the ‘vegan’ package in R software (v4.0.5), the nonmetric multidimensional scaling (NMDS) ordinations based on Bray–Curtis distance matrices was employed to analyzed soil bacterial and fungal community compositions. Using the ‘adonis’ function of the ‘vegan’ package (999 permutations), permutation multivariate analysis of variance (PERMANOVA) was conducted to test for statistically significant differences in the community compositions by forest succession and season. The partial Mantel test with 999 permutations was performed to assess the main drivers that were significantly correlated with the soil bacterial and fungal communities based on Spearman’s correlation (*p* < 0.05) using the “vegan” package.

The phylogenetic normalized stochasticity ratio (pNST) was used to quantify the relative importance of deterministic and stochastic processes in community assembly. The beta nearest taxon indices (βNTI) and Raup-Crick (RC_bray_) null model based on Bray–Curtis dissimilarity were further used to quantify dispersal-based stochastic ecological processes generating the turnover of community composition. For detailed descriptions of the method used, we refer the reader to the study of Ji et al. (2022b). All parameters mentioned above were calculated using the ‘iCAMP’ package in R, with the code provided by Ning et al. (2020).^1^

Co-occurrence networks for the different forest successional stages and seasons were constructed using the molecular ecological network analysis pipeline^2^. We used the Spearman rank correlation to establish co-occurrence networks for the soil bacterial and fungal communities. Then, the same network size and average number of links were employed to generate 100 corresponding random networks. A *Z*-test was performed to test for differences between the empirical network and the random networks. The visualization of soil bacterial and fungal networks was generated by Gephi 0.9.2. For detailed descriptions of the used method, we refer the reader to the studies of Ji et al. (2021, 2022a).

## Results

### Soil physical and chemical properties

Forest succession and season had a significant effect on the soil total P, DOC, DOP, pH and NH_4_^+^-N contents (*p* < 0.05; Table 1). In the wet season, soil C and DOC contents were higher in the late stage (S4) than in the other three stages. Soil NH_4_^+^–N and NO_3_^−^–N contents in the late stage (S4) in the wet season were 190.9 and 117.0% higher than in the initial stage (S1), respectively (*p* < 0.05, Table 1). In the dry season, soil P, DON, and NH_4_^+^–N contents significantly varied with the forest development, but forest succession had no significant effect on the variation of C, N, DOC and DON contents (Table 1). Compared with the wet season, soil DOC content and pH value were strikingly increased in the dry season across the four forest successional stages (*p* < 0.05; Table 1).

**Table 1:** The soil physicochemical property in different stages of forest succession and seasons.

Successional stage	Season	C (g·kg^-1^)	N (g·kg^-1^)	P (g·kg^-1^)	DOC (mg·kg^-1^)	DON (mg·kg^-1^)	DOP (mg·kg^-1^)	pH	NH_4_^+^-N (mg·kg^-1^)	NO_3_^−^-N (mg·kg^-1^)
S1	Wet season	15.99 ± 0.33Bb	8.75 ± 0.2Aab	0.66 ± 0.02Aa	200.41 ± 4.55Ba	43.54 ± 1.94Aa	0.49 ± 0.08Ab	3.89 ± 0.02Bb	11.87 ± 1.08Ab	5.76 ± 1.24Ab
	Dry season	21.09 ± 1.24Aa	9.60 ± 1.06Aa	0.28 ± 0.01Bbc	239.13 ± 5.69Aa	53.48 ± 3.18Aa	0.85 ± 0.17Aa	4.61 ± 0.01Aa	3.11 ± 0.52Bb	13.52 ± 3.08Aa
S2	Wet season	17.37 ± 0.41Ab	9.55 ± 0.16Aa	0.64 ± 0.01Aa	215.90 ± 2.84Bab	54.33 ± 4.29Aa	0.61 ± 0.05Bb	4.11 ± 0.09Bab	30.89 ± 9.19Aa	12.58 ± 5.96Aa
	Dry season	17.36 ± 3.17Aa	11.33 ± 1.19Aa	0.25 ± 0.01Bc	240.50 ± 2.09Aa	53.74 ± 3.66Aa	0.99 ± 0.11Aa	4.66 ± 0.02Aa	28.23 ± 9.35Aa	22.14 ± 3.48Aa
S3	Wet season	22.59 ± 1.04Aa	7.42 ± 0.23Abc	0.38 ± 0.01Ab	214.99 ± 5.13Bab	54.83 ± 5.79Aa	0.43 ± 0.06Bb	4.29 ± 0.09Ba	18.95 ± 3.51Ab	9.04 ± 4.76Aab
	Dry season	16.51 ± 1.67Ba	8.75 ± 1.82Aa	0.36 ± 0.02Aa	245.51 ± 5.97Aa	65.71 ± 3.12Aa	1.09 ± 0.08Aa	4.67 ± 0.04Aa	16.75 ± 4.59Aab	18.66 ± 4.87Aa
S4	Wet season	29.16 ± 0.40Aa	7.30 ± 0.53Bc	0.47 ± 0.05Ab	224.10 ± 5.69Ba	42.84 ± 7.56Aa	1.07 ± 0.10Aa	4.25 ± 0.01Ba	34.53 ± 1.95Aa	12.50 ± 0.60Aa
	Dry season	22.40 ± 0.39Ba	9.99 ± 0.23Aa	0.31 ± 0.01Bb	247.33 ± 4.17Aa	19.63 ± 2.95Bb	1.01 ± 0.19Aa	4.68 ± 0.01Aa	16.36 ± 0.86Bab	14.59 ± 0.71Aa
Two-way ANOVA										
Stage		14.080***	2.584 ns	9.608**	3.950*	16.098***	3.936*	9.76**	7.12**	1.508 ns
Season		3.760 ns	7.045*	244.953***	77.25***	0.057 ns	17.858**	241.687***	4.780*	7.957**
Stage×season		7.799**	0.388 ns	35.869***	1.121 ns	6.495**	3.453*	5.044*	1.050 ns	0.477 ns

S1, initial stage; S2, early stage; S3, middle stage; S4, late stage. NO_3_^−^-N, nitrate nitrogen; NH_4_^+^-N, ammonium nitrogen; DOC, dissolved organic carbon; DON, dissolved organic nitrogen; DOP, dissolved organic phosphorus. Different lowercase letters indicate significant differences based on the Tukey’s HSD test at different forest successional stages in the same season (*p* < 0.05), while different uppercase letters indicate significant differences based on the Student *T*-test in different seasons at the same successional stage. ns, not significant; * *p* < 0.05; ** *p* < 0.01; *** *p* < 0.001.

### General descriptions of sequencing information

After bioinformatic filtering and quality control, 393,260,760 high-quality soil bacterial and 571,964,763 fungal sequences were generated by Illumina MiSeq sequencing across all soil samples. For both bacteria and fungi, the average read lengths were 432 bp and 238 bp, with >99% Good’s coverage for the 16S and ITS gene regions. The rarefaction curves of bacterial and fungal genes approached saturation at 97% sequence similarity, which suggested that the sequencing depth was adequate for evaluating the soil bacterial and fungal structure and diversity across all samples (Supplementary Figure S1).

### Relative abundance and taxonomic composition of soil bacteria and fungi

**Figure 1:**
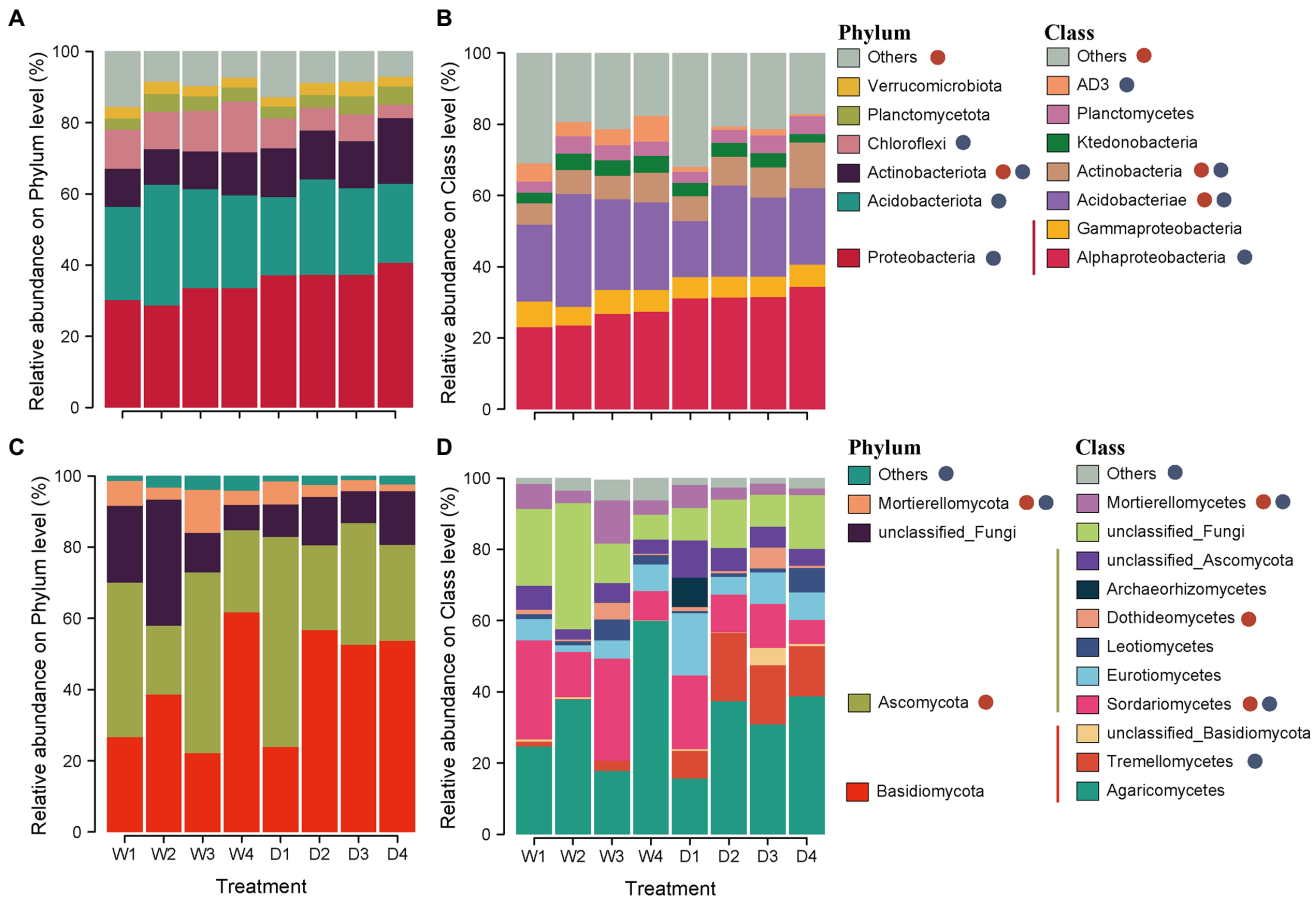
Relative abundances of dominant soil bacterial and fungal phyla **(A,C)** and classes **(B,D)** in different stages of forest succession and seasons. D1, D2, D3 and D4 represent the initial, early, middle, late stages of forest succession in dry season, while W1, W2, W3 and W4 denote the initial, early, middle, late stages of forest succession in wet season. Red and blue circles indicate the significant differences among different stages of forest succession and seasons, respectively.

The 4,187 OTUs were distributed across 31 phyla, 101 classes, and 606 genera. Across all soil samples, the bacterial community was dominated by Proteobacteria (34.8%), Acidobacteriota (26.2%), and Actinobacteriota (12.8%) at the phylum level (Figure 1A). The relative abundance of Actinobacteriota in forest successional stage S4 (D4 and W4) was 62.4% higher than that in forest successional stage S1 (D1 and W1). The relative abundance of Proteobacteria and Actinobacteriota significantly increased in the dry season, while the relative abundance of Acidobacteriot and Chloroflexi showed a higher value in the wet season (Figure 1A). In addition, Alphaproteobacteria, Acidobacteriae and Actinobacteria were the dominant classes, with relative abundances of 28.6, 23.5 and 8.0%, respectively (Figure 1B). Forest succession and season had a significant effect on the relative abundances of Acidobacteriae and Actinobacteria (Figure 1B).

For soil fungi, a total of 4,773 OTUs were identified, distributed among 14 phyla, 42 classes and 558 genera. At the phylum level, the fungal community was dominated by Basidiomycota and Ascomycota, with relative abundances of 42.0 and 35.0%, respectively (Figure 1C). Higher relative abundances of Ascomycota and Mortierellomycota were observed in successional stages S1 and S3, whereas lower values were found in successional stages S2 and S4 (Figure 1C). The dominant fungi at the class level were Agaricomycetes (32.9%) and Sordariomycetes (16.0%) across all soil samples. Forest succession and season both had a significant effect on the relative abundances of Sordariomycetes and Mortierellomycetes (Figure 1D).

### Soil bacterial and fungal diversity in different natural regeneration stages and seasons

**Figure 2:**
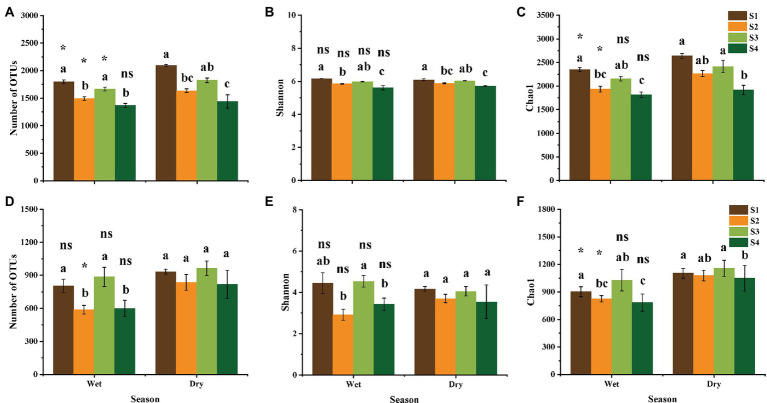
Observed number of OTUs, Shannon and Chao 1 indices of bacterial **(A–C)** and fungal **(D–F)** community in different stages of forest succession and seasons. **(A,D)**, observed number of OTUs; **(B,E)**, Shannon index; **(C,F)**, Chao 1 index. S1, S2, S3, and S4 represent the initial, early, middle, late stages of forest succession, respectively. Different lowercase letters indicate significant differences according to the Tukey’s HSD test at different forest successional stages in the same season (*p* < 0.05). Symbols indicate significant differences according to the Student’s *T* test between different seasons at same successional stage. ns, not significant; *, *p* < 0.05.

Forest succession had a significant effect on soil bacterial and fungal α–diversity (Figures 2A–F). The number of observed OTUs, as well as Shannon and Chao 1 indices of soil bacteria significantly increased from successional stage S1 to S4 (Figures 2A–C), while the α–diversity of soil fungi showed an S-shaped trend (Figures 2D–F). Notably, in the successional stage S2, the observed number of OTUs and Chao 1 index of bacteria and fungi showed a significant decrease in the wet season, compared with those in the dry season (*p* < 0.05; Figures 2A,C,D,F).

**Figure 3:**
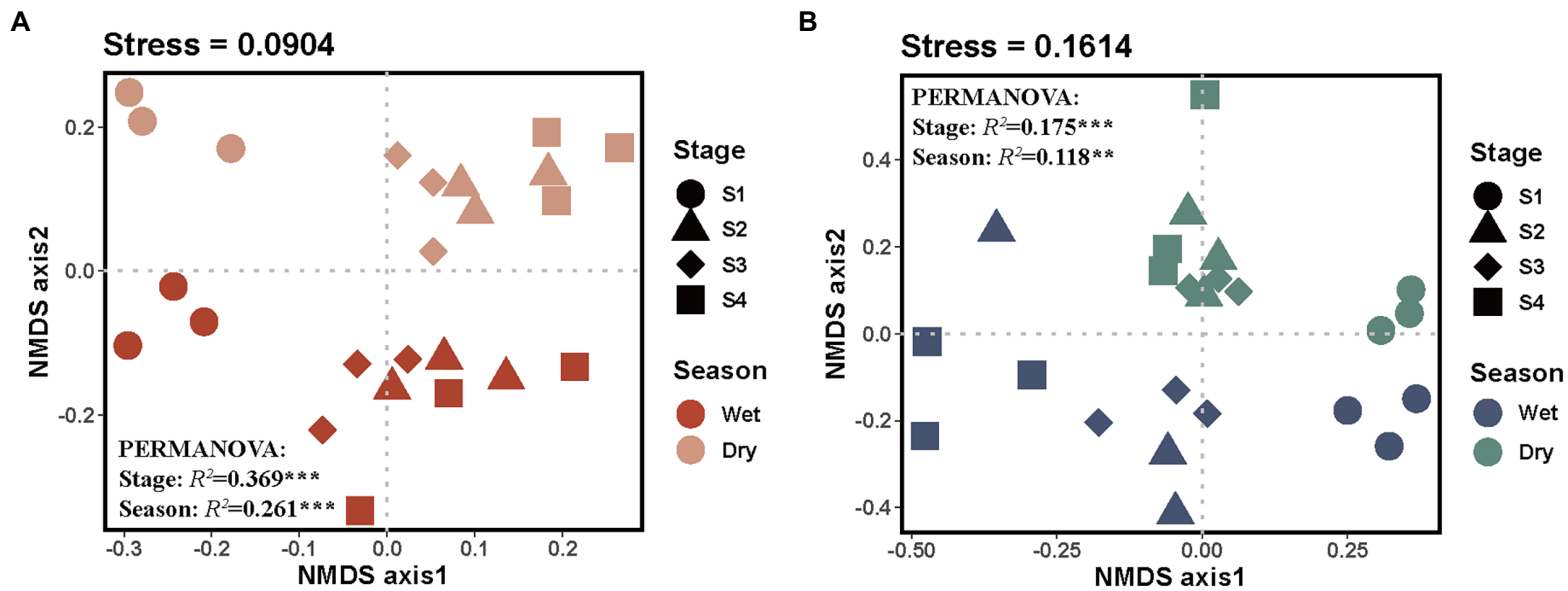
Nonmetric multidimensional scaling ordinations of the Bray–Curtis dissimilarity matrices of bacterial **(A)** and fungal **(B)** communities. S1, initial stage; S2, early stage; S3, middle stage; S4, late stage. Numbers after “Stage” and “Season” are *R*^2^, representing the variation in bacterial and fungal community composition that can be explained by forest succession or season, detected using PERMANOVA. ** and *** denote the significant level at *p* < 0.01 and *p* < 0.001, respectively.

Nonmetric multidimensional scaling (NMDS) analysis was conducted to visualize the differences in soil microbial community composition among different groups (Figure 3). The bacterial and fungal community compositions significantly varied among different stages of forest succession and seasons, and there was a similar pattern for bacterial and fungal communities across all soil samples. Additionally, permutation-based multivariate analysis of variance (PERMANOVA) demonstrated an obvious separation for both bacterial and fungal community compositions (Figures 3A,B). Notably, forest succession had a stronger effect on soil microbial community compositions than season (bacteria, *R*^2^_stage_ = 0.369, *R*^2^_season_ = 0.261; fungi, *R*^2^_stage_ = 0.175, *R*^2^_season_ = 0.118; Figures 3A,B).

### Ecological processes of soil bacterial and fungal community

**Figure 4:**
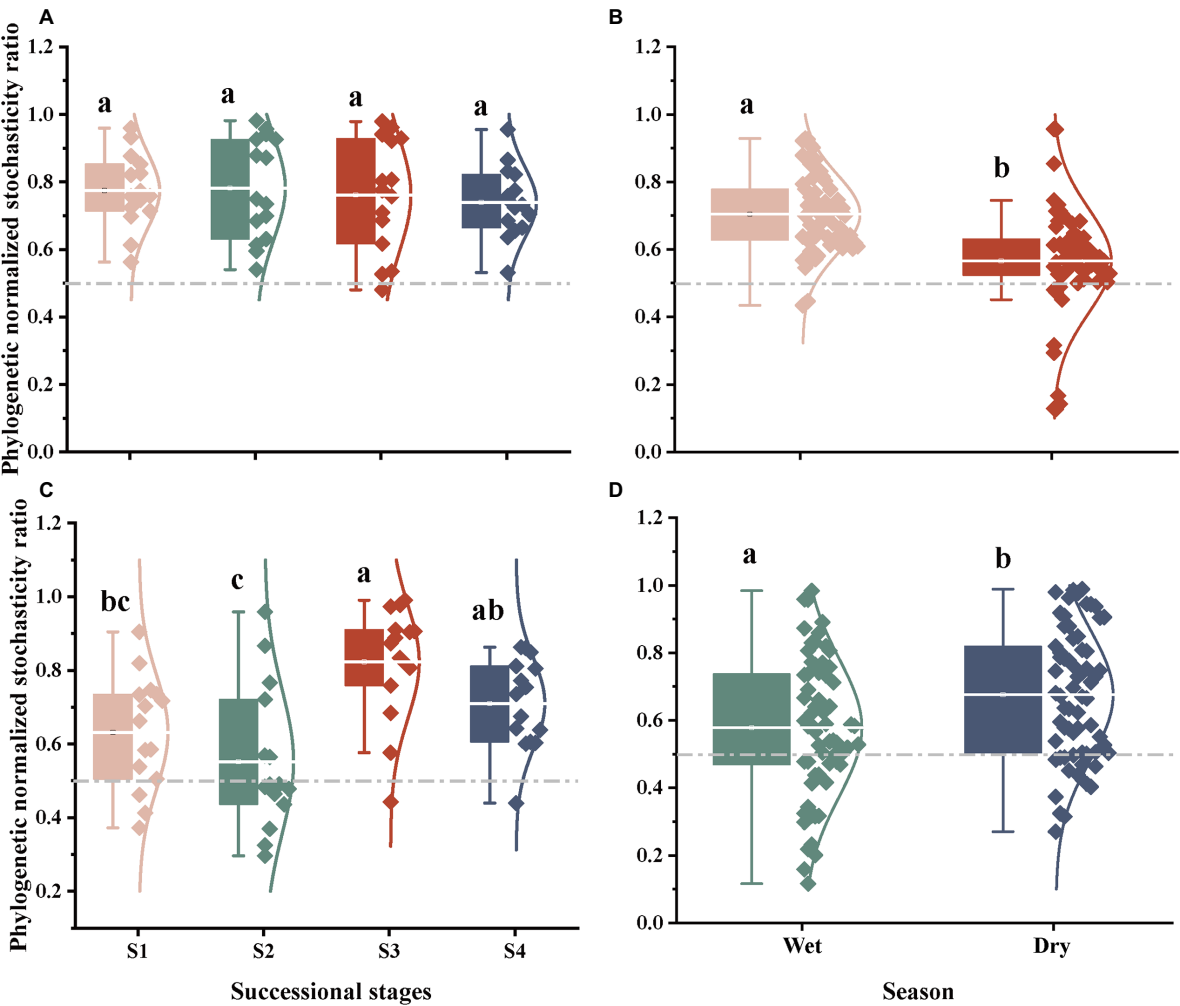
The ecological stochasticity in the potentially bacterial **(A,B)** and fungal **(C,D)** community assembly estimated by the phylogenetic normalized stochasticity ratio (pNST). **(A)**, ecological stochasticity of soil bacteria among different stages of forest succession; **(B)**, ecological stochasticity of soil bacteria between different seasons. **(C)**, ecological stochasticity of soil fungi among different stages of forest succession; **(D)**, ecological stochasticity of soil fungi between different seasons. The value of 0.5 as the boundary point between more deterministic (<0.5) and more stochastic (>0.5) assembly. S1, initial stage; S2, early stage; S3, middle stage; S4, late stage. Different lowercase letters indicate significant differences in different stages of forest succession (or different season) (*p* < 0.05). Differences between dry and wet seasons were examined using a Student’s *T*-test. Differences among different stages of forest succession were examined using the Tukey’s HSD test.

**Figure 5:**
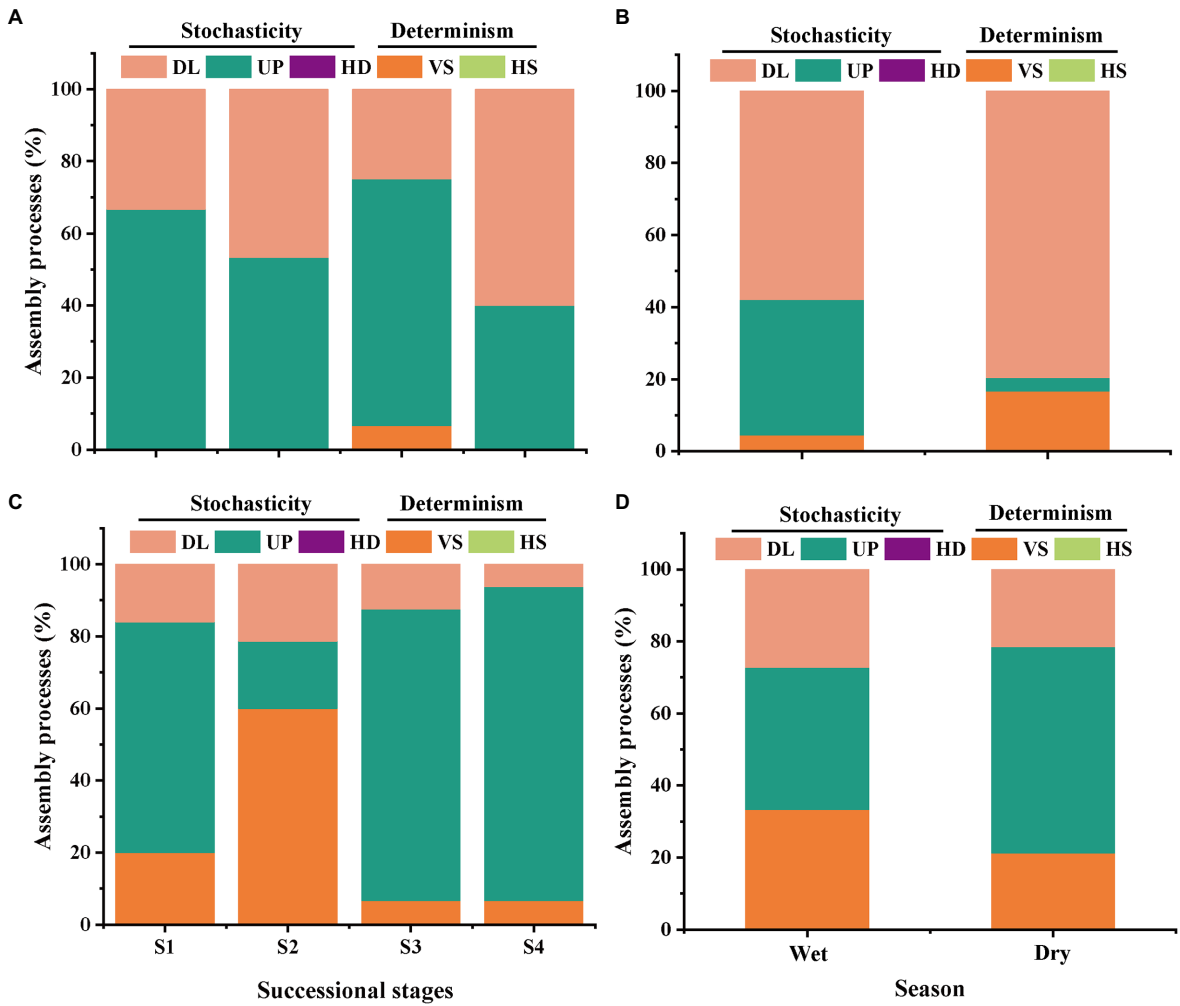
The relative contributions (%) of the community assembly processes in shaping soil bacterial **(A,B)** and fungal **(C,D)** communities in different stages of forest succession and seasons. HS, homogeneous selection; *VS*, variable selection; HD, homogenizing dispersal; UP, undominated process; DL, dispersal limitation. S1, initial stage; S2, early stage; S3, middle stage; S4, late stage.

Based on the taxonomic and phylogenetic metrics, the phylogenetic normalized stochasticity ratio (pNST) was calculated, in order to assess the ecological processes associated to bacterial and fungal community assembly (Figures 4, 5). Season had a significant effect on the bacterial and fungal community assembly, while forest succession only affected the variation of soil fungal pNST value (Figures 4B,D,C). Meanwhile, the ecological processes of both bacteria and fungi were primarily dominated by stochasticity (Figure 5). In stage S4, a high proportion of drift and dispersal limitations were observed for soil bacterial and fungal communities (Figures 5A–D). In addition, the community assembly of bacteria and fungi exhibited a contrasting pattern in dry and wet seasons; that is, increasing and decreasing variable selection in the dry season, respectively (Figures 5B,D).

### Main driving factors of soil microbial community composition

**Figure 6:**
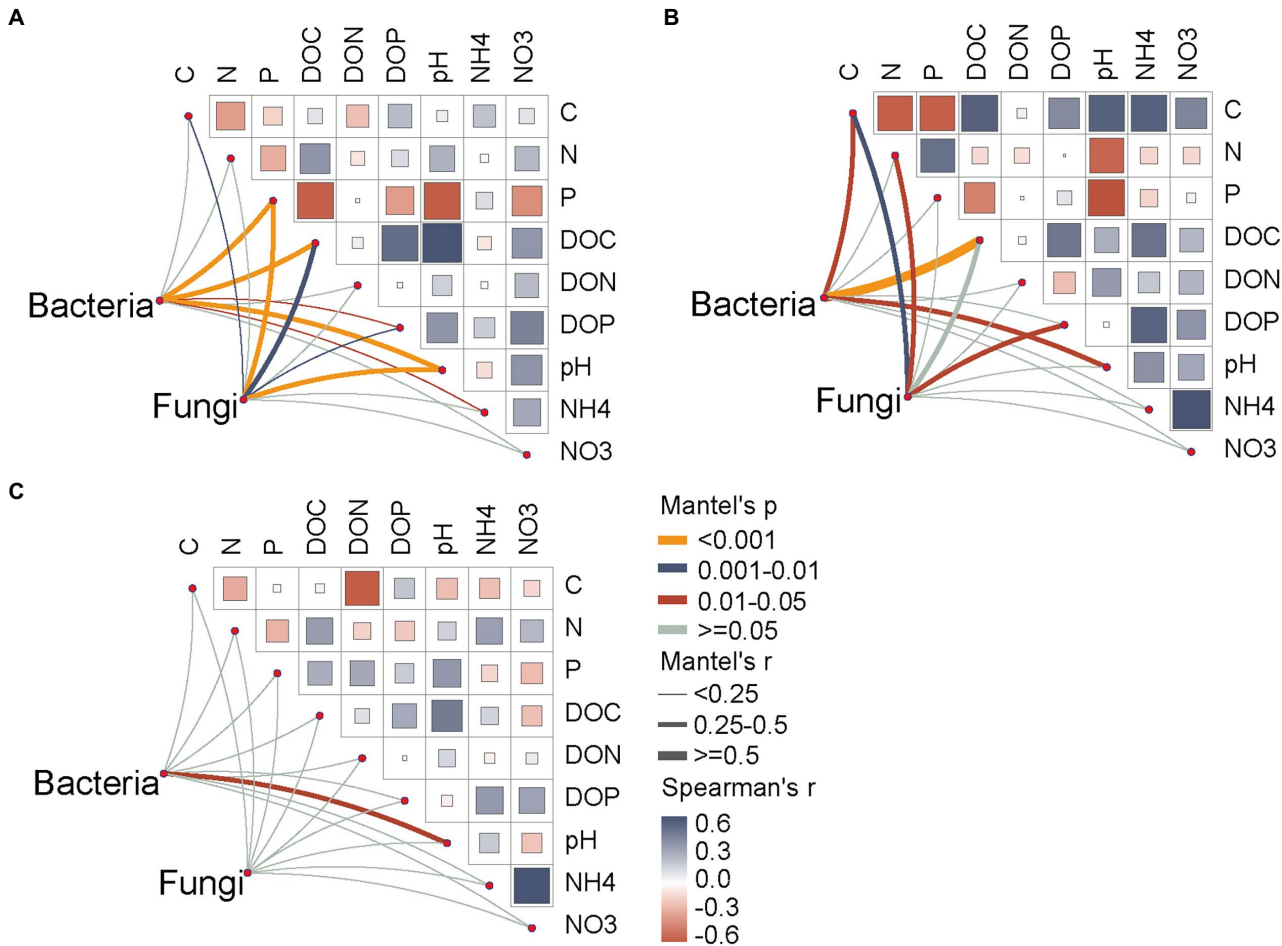
Total and dissolved organic drivers of the soil bacterial and fungal community compositions. **(A)** all seasons; **(B)** wet season; **(C)** dry season. Soil microbial community composition was correlated to soil variables by partial Mantel tests based on the Bray–Curtis distance. Pairwise comparisons of total and dissolved organic parameters are shown at the upper-right, with a color gradient representing Spearman’s correlation coefficients. The edge width represents the partial Mantel’s r statistic for the corresponding correlation, and edge color denotes that significance are tested based on 999 permutations. NO3, nitrate nitrogen; NH4, ammonium nitrogen; DOC, dissolved organic carbon; DON, dissolved organic nitrogen; DOP, dissolved organic phosphorus.

Soil total P, DOC, DOP and pH were significantly correlated with the soil bacterial and fungal community compositions across all seasons (Figure 6A). In the wet season, the soil bacterial community was affected by soil carbon-related variables (C and DOC) and pH, whereas the fungal community changed with the variations in soil total C, N, and DOP contents (Figure 6B). Notably, there were no significant relationships between soil bacterial communities and soil factors (excluding soil pH) in the dry season (Figure 6C).

### Co-occurrence networks of soil bacterial and fungal communities during forest successions

**Figure 7:**
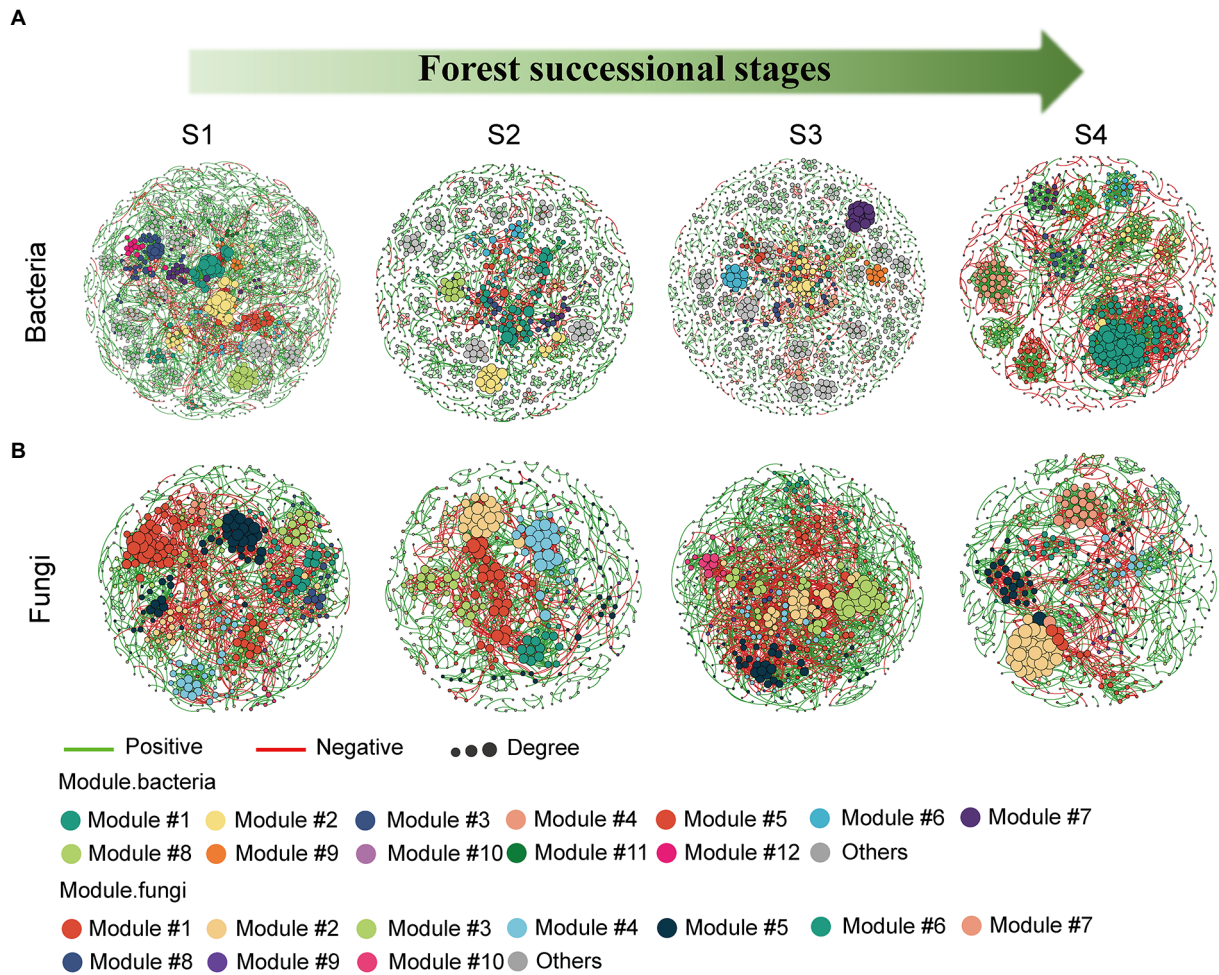
Modular networks of soil bacteria **(A)** and fungi **(B)** during different forest successions. Node colors represent different modules. The connections denote a strong (Spearman’s ρ > 0.6) and significant (*p* < 0.01) correlations. S1, initial stage; S2, early stage; S3, middle stage; S4, late stage.

Molecular ecological networks for soil bacteria and fungi were constructed, in order to explore the effect of forest succession on the microbial interactions in both seasons (Figure 7, Supplementary Figure S2). Forest succession and season both had obvious effects on the topological properties of bacterial and fungal networks (Tables 2, 3). The average degree of soil microbial network showed a decrease in the early stage and increase in the middle and late stages of forest succession, suggesting that more complex networks of both bacteria and fungi are induced by forest succession (Tables 2, 3). Additionally, we observed an increasing proportion of negative links and positive links in soil bacterial and fungal networks with progressive forest succession, respectively, which indicated more competition and mutualism in the late stages (Figures 7A,B; Tables 2, 3). However, we also observed a slight increase in the proportion of negative links in soil microbial networks in the dry season as compared with the wet season (Supplementary Figure S2). The number of bacterial and fungal keystones was higher in the dry season than in the wet season (Supplementary Figure S3; Supplementary Tables S1, S2).

**Table 2:** Topological properties of the taxonomic network in soil bacteria.

Network	Topological properties	Forest successional stages				Seasons	
		S1	S2	S3	S4	Wet	Dry
Empirical network	Number of nodes	1,457	1,086	1,292	767	706	671
	Number of links	3,501	1770	2,279	2,950	1955	2093
	*R*^2^ of power-law	0.73	0.813	0.803	0.787	0.912	0.879
	Number of positive correlations	2,890 (82.5%)	1,346 (76.0%)	1794 (78.7%)	1,149 (38.9%)	1903 (97.3%)	1,680 (80.6%)
	Number of negative correlations	611 (17.5%)	424 (24.0%)	485 (21.3%)	1801 (61.1%)	52 (2.7%)	407 (19.4%)
	Average degree (avgK)	4.806	3.26	3.528	7.692	5.538	6.238
	Average clustering coefficient (avgCC)	0.628	0.589	0.58	0.258	0.299	0.199
	Average path distance (GD)	8.166	8.373	6.774	8.785	9.307	4.746
	Modularity	0.955	0.958	0.963	0.74	0.762	0.485
Random network	avgCC±SD	0.009 ± 0.002	0.005 ± 0.002	0.005 ± 0.002	0.030 ± 0.003	0.025 ± 0.003	0.050 ± 0.005
	GD ± SD	4.347 ± 0.021	5.379 ± 0.044	5.178 ± 0.033	3.421 ± 0.018	3.746 ± 0.030	3.492 ± 0.028
	Modularity±SD	0.450 ± 0.004	0.603 ± 0.004	0.569 ± 0.004	0.311 ± 0.004	0.392 ± 0.004	0.344 ± 0.004

S1, initial stage; S2, early stage; S3, middle stage; S4, late stage.

**Table 3:** Topological properties of the taxonomic network in soil fungi.

Network	Topological properties	Forest successional stages				Seasons	
		S1	S2	S3	S4	Wet	Dry
Empirical network	Number of nodes	596	496	689	507	287	344
	Number of links	2,079	1,557	2,365	2,152	482	663
	*R*^2^ of power-law	0.654	0.645	0.735	0.624	0.919	0.926
	Number of positive correlations	1,210 (58.2%)	1,020 (65.5%)	1,320 (55.8%)	1,544 (71.7%)	371 (77.0%)	479 (72.2%)
	Number of negative correlations	869 (41.8%)	537 (34.5%)	1,045 (44.2%)	608 (28.3%)	111 (23.0%)	184 (27.8%)
	Average degree (avgK)	6.977	6.278	6.865	8.489	3.359	3.855
	Average clustering coefficient (avgCC)	0.48	0.523	0.486	0.562	0.151	0.188
	Average path distance (GD)	6.634	6.434	6.3	7.087	5.669	4.657
	Modularity	0.789	0.753	0.756	0.734	0.658	0.648
Random network	avgCC ± SD	0.022 ± 0.003	0.034 ± 0.005	0.026 ± 0.003	0.062 ± 0.005	0.023 ± 0.006	0.024 ± 0.006
	GD ± SD	3.493 ± 0.020	3.477 ± 0.031	3.518 ± 0.022	3.137 ± 0.022	4.109 ± 0.073	3.978 ± 0.052
	Modularity ± SD	0.342 ± 0.004	0.359 ± 0.004	0.344 ± 0.004	0.285 ± 0.004	0.550 ± 0.007	0.505 ± 0.007

S1, initial stage; S2, early stage; S3, middle stage; S4, late stage.

## Discussion

### Effect of forest succession on soil microbial communities is more pronounced than the effect of season

Our results supported the first hypothesis that soil fungi exhibit a more sensitive association with forest succession compared with bacteria. The dominant fungal phyla changed more frequently than the dominant bacterial phyla, and there was an obvious shift in soil fungal taxa and their relative abundance along the forest successional gradient (Figure 1). Generally, due to the high growth and turnover rates, soil bacteria dominated at the early stage of forest development, while soil fungi, with a low turnover rate, tend to predominate in the late stages (Fierer et al., 2007; Zhou et al., 2017). Additionally, plant species-specific effects were considered to have a greater impact on the fungal community (Hannula et al., 2019). Soil bacteria tend to present much greater species richness than fungi in the same habitat; therefore, the bacteria are likely to be better adapted to non-extreme perturbations due to their high diversity (He et al., 2017). Previous studies have demonstrated that Proteobacteria and Acidobacteriota include fast-growing copiotrophs and slow-growing oligotrophs, respectively, and Proteobacteria are inclined to carbon-rich conditions (Fierer et al., 2007; Zechmeister-Boltenstern et al., 2015). In our study, the relative abundance of Proteobacteria and Acidobacteriota increased and decreased with forest successional series, respectively (Figure 1A), which is consistent with the results of Ren et al. (2019). A potential explanation is that the growth of Proteobacteria and Acidobacteriota may be related to the increasing soil organic carbon content and limitation of nutrients with forest succession. Our previous study confirmed that *P. bournei*-dominated forests became progressive limited in soil N and P contents from the initial stage to late stage of forest succession (Wang et al., 2021). In addition, we observed that the relative abundances of Proteobacteria, Actinobacteriota, Acidobacteriota, and Chloroflexota significantly varied between dry and wet seasons. Seasonal fluctuation of different phyla is related to carbon substrate supply due to root exudation and plant litter among seasons (Lipson, 2007; Kuffner et al., 2012). As a proxy for other variables along environmental gradients, soil pH influences microbial growth and metabolism (Malik et al., 2017). Dissolved organic substrates (DOC, DON and DOP) and soil pH exhibited an obvious variation over time in our study, and presented a strong association with bacterial community in the wet season (Figure 6B), further supporting our findings. Meanwhile, the relative abundance of Basidiomycota increased with forest succession (Figure 1C), which is in line with the results reported by Liu et al. (2020). Although the vegetation composition was not surveyed in the present study, there is a consensus that soil fungal community is closely associated with plant community attributes along the progression of succession (Blaalid et al., 2012; Zechmeister-Boltenstern et al., 2015).

In addition, both bacterial and fungal beta diversity were detected to have massive shifts in community compositions along a forest successional trajectory. Compared with the season, the forest successional stage had a stronger effect on soil microbial community composition (Figure 3), which can be ascribed to the variation in soil pH and soil dissolved nutrients (Cline and Zak, 2015). Recent studies have revealed that fast-changing environmental factors (e.g., dissolved organic nutrients) influence the seasonal variation of soil microbial communities, whereas slow-changing soil properties (e.g., pH and soil total elements) played important roles over large spatial scales or longer periods (Zhang et al., 2020; Ji et al., 2021). In the current study, the partial Mantel test showed that soil pH significantly affected the soil bacterial and fungal communities in both dry and wet seasons, while soil dissolved organic nutrients only affected the composition of soil microbes in the wet season (Figure 6). Soil pH is a critical factor that affects the structure of soil bacterial and fungal communities (Fierer and Jackson, 2006). As succession proceeds, increasing soil pH resulted in greater OTU richness, while deviations in soil pH may impose stress on single-celled organisms and limit the survival of taxa outside of their optimal pH range (Fierer and Jackson, 2006). Nonetheless, caution should be taken when interpreting our results, as only two sampling time points were included in this study. Collectively, our results provide solid empirical evidence that forest succession has a stronger effect on soil microbial community composition than season.

### Contrasting response of soil bacterial and fungal community assembly processes to forest succession and season

Accumulating evidence has demonstrated that the soil microbial community is primarily shaped by niches *v.s.* neutral processes (Stegen et al., 2015; Zhou and Ning, 2017; Ji et al., 2022b). In addition to determinism (e.g., environmental selection and filtering), stochastic processes are considered as another primary force governing soil microbial community assembly in forest ecosystems (Stegen et al., 2015). In the present study, we observed an increased relative contribution of stochastic processes in soil fungal community across the forest successional gradient, whereas soil bacterial community did not present such a response (Figure 5). This finding supports our second prediction: that soil bacterial community assemblages respond differently to forest succession, compared to the fungi community assemblages. Dini-Andreote et al. (2014) have revealed that, as succession proceeds, phylogenetic turnover of soil bacteria is reduced as a result of the increased soil buffer effect, more prominent plant communities, and decreased variation in amplitudes. Thus, our findings imply that the soil bacteria may reach a relatively stable and modest status in the late stage of forest succession, and the bacterial species tend to interact more with each other (or among kingdoms) than they do with the local environments (Liu et al., 2020). Our prior study provided evidence of soil N and P limitation in the late succession stage of *P. bournei*-dominated forests (Wang et al., 2021). At the late climax of forest succession, poor substrate quality (e.g., low N availability) is generally associated with aggressive fungal colonization, as well as enzyme activities that decelerate the decomposition of litter (Waring et al., 2016). Previous studies have reported that drift is more prevalent when populations are small and/or diversity is lower (Chase and Myers, 2011). Limited nutrient availability may induced the low diversity of soil fungi, which can provide a potential explanation for the observed increase in stochasticity of fungal community assembly with forest succession.

The patterns of community assembly in seasonal variability between soil bacteria and fungi were also markedly different, with increased proportion of determinism and stochasticity for bacteria and fungi from the wet to dry season, respectively (Figure 5), which could be attributed to seasonal variations in litter inputs and microclimate conditions. Several recent analyses have proven that the proportion of deterministic processes likely increases with plant richness, due to the positive feedback in soil microbes and between plants and microbes caused by high plant richness and available resources (Ning et al., 2020; Liu et al., 2021). There is a general consensus that plant community changes are significant for fungal communities, but less relevant for bacterial communities during forest succession (Williams et al., 2013; Cutler et al., 2014). These results may partly explain the higher proportion of variable selection in soil fungal community assembly than in soil bacteria. Moreover, soil bacteria turnover times have been generally estimated to be 10-fold faster than fungi (Bååth, 1998); thus, a higher proportion of dispersal limitation can be observed in the soil bacterial community. Consequently, the presented results provide evidence that there is an obvious shift in relative contribution of community assembly of soil bacteria and fungi with forest succession.

### Complexity of soil microbial networks increased with forest succession

As stated in our third hypothesis, increasing co-occurrence network complexity of soil bacteria and fungi was observed along with increasing forest development, with higher connectivity in the latter stages of succession (Figure 7). This result is consistent with that of Sun et al. (2017), implicating stronger coupling associations between microbes in mediating soil carbon and nitrogen cycles in the late stage of forest succession. As described previously, the secondary succession of forests promotes the formation of dense root systems and increases nutrient release rates, which stimulates soil fungal activity, resulting in a more complex fungal community structure and enhanced environmental resilience (Liang et al., 2017; Hu et al., 2022). Previous studies have found that microbial networks with high modularity, and less but larger modular communities tend to be more stable (Chen et al., 2022; Liu et al., 2022). In our study, the high modularity of bacterial and fungal networks was simultaneously observed in the initial stage of forest succession, indicating that the microbial communities became more volatile with forest succession (Tables 2, 3). More interestingly, in addition to increased network complexity, the soil bacteria and fungi exhibited contrasting strategies; that is, more competition and cooperation, respectively, along the forest successional gradient (Tables 2, 3). Generally, positive interactions reflect cooperation and niche differentiation among species in the networks, whereas negative interactions indicate competition and niche overlap among species (Ghoul and Mitri, 2016). These results were probably due to the latter forest successional stage being composed of various tree species, which provided diverse substrates for soil fungi and resulted in a greater mutualism in participating organic matter decomposition, nutrient circulation, and complex networks. Despite the improved metabolic efficiency induced by increased cooperation within communities, this can also compromise ecological stability (Coyte et al., 2015; Hernandez et al., 2021). On the other hand, as succession proceeds, soil C and N substrate availabilities usually increase and the late stage of forest succession is characterized by nutrient-rich conditions (Waring et al., 2016), which may enhance competition among bacterial species. As succession proceeds, the greater amount of nutrients will stimulate bacteria growth, resulting in more competition for nutrients (Zhao et al., 2016). Moreover, it is generally considered that soil fungi dominate the microbial community at the later stage of forest succession, which will compete for soil resources with bacteria (Fierer et al., 2007; Zhou et al., 2017).

Further, habitat conversion can reshape the distinct key taxa within these co-occurrence networks, resulting in changes in their interactions (Ji et al., 2021). Additionally, network complexity and stability can lead to a similar trend in keystone taxa numbers, suggesting that more keystone taxa constitute a more stable network (Chen et al., 2022). Compared with the wet season, more bacterial (8 module hubs, 17 connectors) and fungal (8 module hubs, 15 connectors) taxa were found in the dry season (Supplementary Figure S2), including Xanthobacteraceae, *Bryobacter* and *Mortierella*, among others. It is well-known that Xanthobacteraceae are potentially involved in nitrogen fixation (Oren, 2014), and *Mortierella* are soil saprophytes that feed on soil organic matter and usually form ectomycorrhizae with plants (Hu et al., 2022). In addition, some *Mortierella* members dissolve inorganic phosphorus effectively in the soil and secrete oxalic acid (Osorio and Habte, 2001), which assists plants and mycorrhizal fungi in absorbing phosphorus. The genus *Bryobacter* accommodates acidotolerant, strictly aerobic, and slow-growing chemo-organotrophic bacteria (Kulichevskaya et al., 2010). Taken together, our results confirm that forest succession leads to an obvious disruption of bacterial and fungal networks, generating contrasting network features between soil bacterial and fungal communities.

## Conclusion

The intact secondary succession in *P. bournei*-dominated forests in the subtropical region provides a unique dynamic landscape for the investigation of patterns in microbial community succession. The results presented in this paper highlight the numerous ways in which the different properties of soil bacteria and fungi can influence their variability during forest succession both in dry and wet seasons. First, our results demonstrated that soil fungi were more sensitive than bacteria along the successional gradient, especially in the aspects of community composition and assembly. More specifically, soil bacterial and fungal communities were primarily dominated by dispersal limitation and drift processes, respectively. Additionally, as succession proceeded, more complex networks of both taxa were found in the latter stages. Soil bacterial (more competition) and fungal (more cooperation) communities exhibited a contrasting pattern over forest succession. Our findings have important implications for understanding how complex soil microbial communities dwelling in *P. bournei*-dominated forests respond to forest succession and season. Further investigations focus on the ecological functions of specific taxa are warranted, which is expected to be essential in order to obtain a better understanding of the potential functions of the microbiota during forest succession. Meanwhile, more compartments and niches should be taken into account in future research, in order to provide a more comprehensive view of microbial ecology along the forest successional trajectories.

## Data availability statement

The raw sequences data was deposited in National Center for Biotechnology Information (NCBI) with Sequence Read Archive (SRA) database under BioProject ID: PRJNA870323 (bacteria) and PRJNA870328 (fungi).

## Author contributions

GH and LJ conceptualized the experiment. YG and TP conducted the field work. TP and SW collected data. LJ and TP performed the data analyses. LJ, ZL, and GH wrote the manuscript and revised the manuscript. All authors commented and contributed to the final version of the manuscript.

## Funding

This work was financially supported by the Natural Science Foundation of China (32071752) and the National Key Research and Development Program of China (2021YFD2201303).

## Conflict of interest

The authors declare that the research was conducted in the absence of any commercial or financial relationships that could be construed as a potential conflict of interest.

## Publisher’s note

All claims expressed in this article are solely those of the authors and do not necessarily represent those of their affiliated organizations, or those of the publisher, the editors and the reviewers. Any product that may be evaluated in this article, or claim that may be made by its manufacturer, is not guaranteed or endorsed by the publisher.

## Footnotes

^1^ https://github.com/DaliangNing/iCAMP1

^2^ http://ieg4.rccc.ou.edu/mena/
