# Supplementary material for: Forest succession improves the complexity of soil microbial interaction and ecological stochasticity of community assembly: Evidence from Phoebe bournei-dominated forests in subtropical regions
Source: Front Microbiol. 2022 Nov 28;13:1021258. doi: 10.3389/fmicb.2022.1021258 (PMC9742230; doi:10.3389/fmicb.2022.1021258)
Supplement: Supplementary file 1 [file Data_Sheet_1.docx]

**Supplementary materials**

**Original research**

**Title: Forest succession improves the complexity of soil microbial interaction and ecological stochasticity of community assembly: Evidence from *Phoebe bournei*-dominated forests in subtropical regions**

**Gongxiu He, Tieshuang Peng, Yi Guo, Shizhi Wen, Li Ji^*^, Zhong Luo^*^**

School of Forestry, Central South University of Forestry and Technology, 410004 Changsha, P.R. China;

*Corresponding author:

Zhong Luo, [luozhong@csuft.edu.cn](mailto:luozhong@csuft.edu.cn)

Li Ji (Correspondence), [jlnefu@hotmail.com](mailto:jlnefu@hotmail.com), School of Forestry, Central South University of Forestry and Technology, 410004 Changsha, P.R. China

**Running title:** Successional patterns of soil microbial community

**Number of words:** 5137 words

**Number of Tables:** 3

**Number of Figures:** 7

**Number of references:** 58


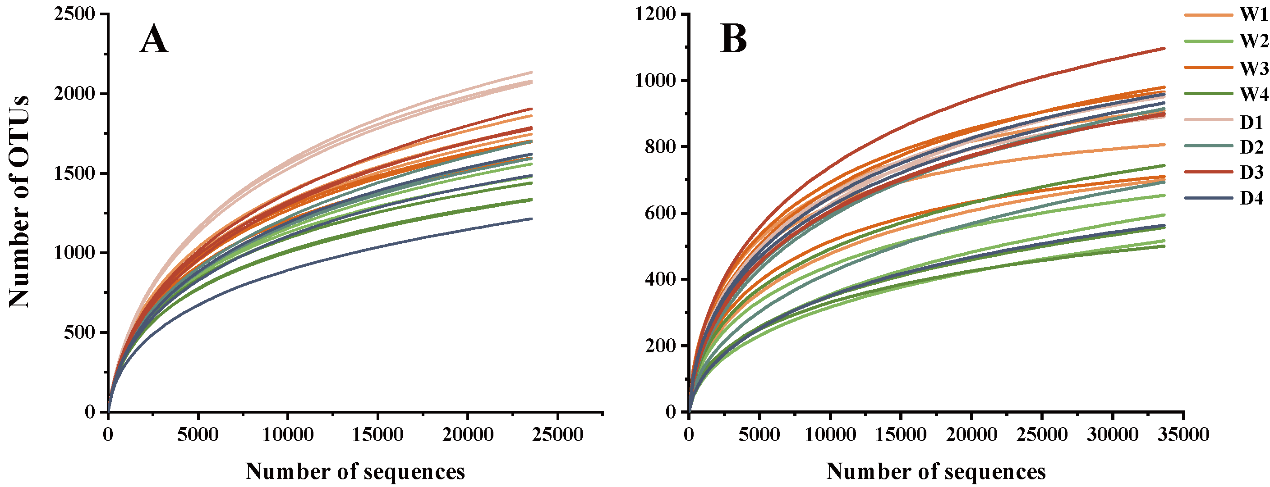


**Figure S1 The rarefaction curves of the number of operational taxonomic units (OTUs) for soil bacterial (A) and fungal (B) communities.**


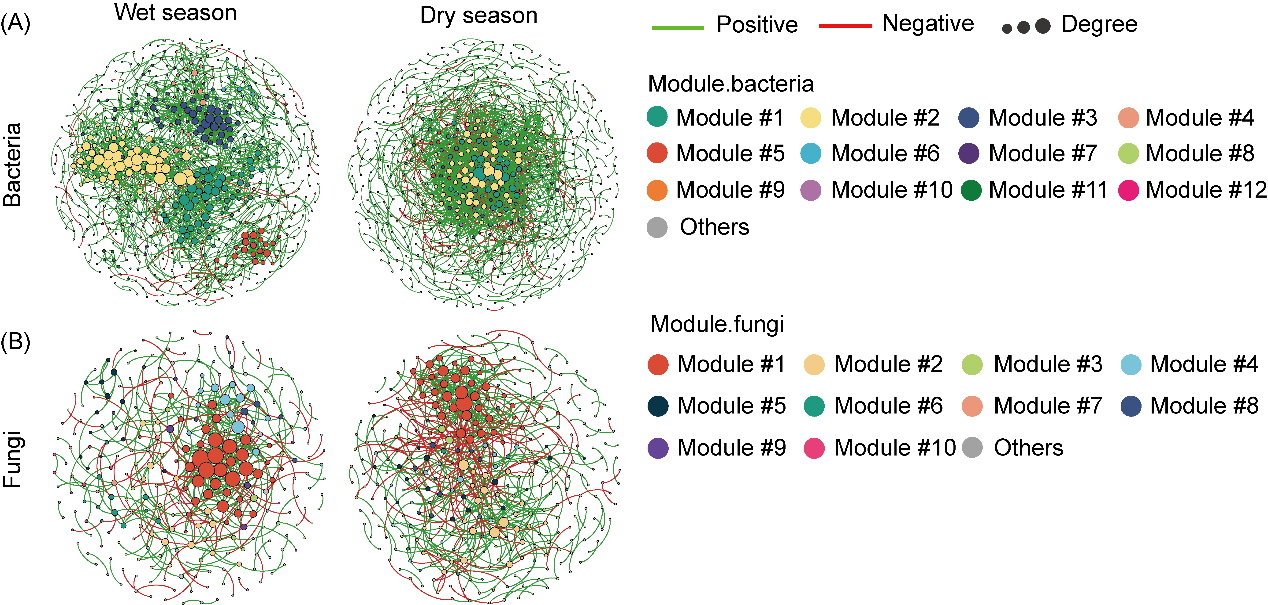


**Figure S2 Modular networks of soil bacteria (A) and fungi (B) during different seasons.** Node colors represent different modules. The connections denote a strong (Spearman’s ρ > 0.6) and significant (*P* < 0.01) correlations.

**Table S1 The taxonomic composition of keystone in soil bacterial community**

| Group | OTU ID | Topological role | Phylum | Class | Order | Family | Genus | Ecological function |
| --- | --- | --- | --- | --- | --- | --- | --- | --- |
| S1 | OTU1882 | Connector | Bdellovibrionota | Oligoflexia | 0319-6G20 | norank_o__0319-6G20 | norank_f__norank_o__0319-6G20 | —— |
|  | OTU343 | Connector | Chloroflexi | Ktedonobacteria | Ktedonobacterales | Ktedonobacteraceae | JG30a-KF-32 | chemoheterotrophy |
|  |  |  |  |  |  |  |  |  |
| S2 | OTU249 | Module hub | Gemmatimonadota | Gemmatimonadetes | Gemmatimonadales | Gemmatimonadaceae | norank_f__Gemmatimonadaceae | chemoheterotrophy |
|  | OTU2471 | Module hub | Chloroflexi | Ktedonobacteria | Ktedonobacterales | JG30-KF-AS9 | norank_f__JG30-KF-AS9 | —— |
|  | OTU82 | Module hub | Acidobacteriota | Vicinamibacteria | Vicinamibacterales | norank_o__Vicinamibacterales | norank_f__norank_o__Vicinamibacterales | —— |
|  | OTU2549 | Connector | Proteobacteria | Gammaproteobacteria | Gammaproteobacteria_Incertae_Sedis | unclassified_o__Gammaproteobacteria_Incertae_Sedis | Candidatus_Berkiella | —— |
|  |  |  |  |  |  |  |  |  |
| S3 | OTU325 | Module hub | Proteobacteria | Gammaproteobacteria | Salinisphaerales | Solimonadaceae | Nevskia | chemoheterotrophy |
|  | OTU3467 | Module hub | Nitrospirota | Nitrospiria | Nitrospirales | Nitrospiraceae | Nitrospira | —— |
|  | OTU830 | Module hub | Chloroflexi | Anaerolineae | norank_c__Anaerolineae | norank_o__norank_c__Anaerolineae | norank_f__norank_o__norank_c__Anaerolineae | —— |
|  | OTU2783 | Module hub | Firmicutes | Clostridia | Clostridiales | Caloramatoraceae | Fonticella | —— |
|  | OTU1292 | Module hub | Gemmatimonadota | Gemmatimonadetes | Gemmatimonadales | Gemmatimonadaceae | Gemmatimonas | —— |
|  | OTU1160 | Module hub | NB1-j | norank_p__NB1-j | norank_c__norank_p__NB1-j | norank_o__norank_c__norank_p__NB1-j | norank_f__norank_o__norank_c__norank_p__NB1-j | —— |
|  |  |  |  |  |  |  |  |  |
| S4 | OTU3237 | Module hub | Firmicutes | Clostridia | Lachnospirales | Lachnospiraceae | Herbinix | —— |
|  | OTU899 | Module hub | Actinobacteriota | Actinobacteria | Frankiales | Acidothermaceae | Acidothermus | aerobic_chemoheterotrophy |
|  | OTU2697 | Module hub | Bacteroidota | Bacteroidia | Chitinophagales | Chitinophagaceae | Dinghuibacter | —— |
|  | OTU2053 | Module hub | Chloroflexi | Ktedonobacteria | Ktedonobacterales | Ktedonobacteraceae | norank_f__Ktedonobacteraceae | —— |
|  | OTU398 | Module hub | Myxococcota | Myxococcia | Myxococcales | Myxococcaceae | norank_f__Myxococcaceae | chemoheterotrophy |
|  | OTU3406 | Module hub | Chloroflexi | Ktedonobacteria | Ktedonobacterales | JG30-KF-AS9 | norank_f__JG30-KF-AS9 | —— |
|  | OTU1629 | Module hub | Firmicutes | Bacilli | Bacillales | Bacillaceae | Bacillus | —— |
|  | OTU2134 | Module hub | Proteobacteria | Gammaproteobacteria | Coxiellales | Coxiellaceae | Coxiella | human_pathogens_all |
|  | OTU3330 | Module hub | Myxococcota | Polyangia | Polyangiales | Polyangiaceae | Pajaroellobacter | —— |
|  | OTU256 | Connector | Armatimonadota | Fimbriimonadia | Fimbriimonadales | Fimbriimonadaceae | norank_f__Fimbriimonadaceae | intracellular_parasites |
|  | OTU1627 | Connector | Elusimicrobiota | Lineage_IIa | norank_c__Lineage_IIa | norank_o__norank_c__Lineage_IIa | norank_f__norank_o__norank_c__Lineage_IIa | —— |
|  |  |  |  |  |  |  |  |  |
| Wet season | OTU3158 | Module hub | Proteobacteria | Alphaproteobacteria | Sphingomonadales | Sphingomonadaceae | Sphingomonas | aerobic_chemoheterotrophy |
|  | OTU3492 | Module hub | GAL15 | norank_p__GAL15 | norank_c__norank_p__GAL15 | norank_o__norank_c__norank_p__GAL15 | norank_f__norank_o__norank_c__norank_p__GAL15 | —— |
|  | OTU2034 | Module hub | Verrucomicrobiota | Chlamydiae | Chlamydiales | Parachlamydiaceae | Candidatus_Protochlamydia | intracellular_parasites |
|  | OTU1089 | Module hub | Acidobacteriota | Blastocatellia | 44889 | norank_o__11-24 | norank_f__norank_o__11-24 | —— |
|  | OTU150 | Module hub | Myxococcota | Myxococcia | Myxococcales | Myxococcaceae | norank_f__Myxococcaceae | intracellular_parasites |
|  | OTU2750 | Module hub | Proteobacteria | Gammaproteobacteria | Burkholderiales | Nitrosomonadaceae | Ellin6067 | —— |
|  | OTU3176 | Module hub | WPS-2 | norank_p__WPS-2 | norank_c__norank_p__WPS-2 | norank_o__norank_c__norank_p__WPS-2 | norank_f__norank_o__norank_c__norank_p__WPS-2 | —— |
|  | OTU919 | Connector | Proteobacteria | Gammaproteobacteria | Burkholderiales | Comamonadaceae | unclassified_f__Comamonadaceae | —— |
|  |  |  |  |  |  |  |  |  |
| Dry season | OTU1740 | Module hub | Chloroflexi | TK10 | norank_c__TK10 | norank_o__norank_c__TK10 | norank_f__norank_o__norank_c__TK10 | —— |
|  | OTU2911 | Module hub | Actinobacteriota | Acidimicrobiia | IMCC26256 | norank_o__IMCC26256 | norank_f__norank_o__IMCC26256 | —— |
|  | OTU2124 | Module hub | RCP2-54 | norank_p__RCP2-54 | norank_c__norank_p__RCP2-54 | norank_o__norank_c__norank_p__RCP2-54 | norank_f__norank_o__norank_c__norank_p__RCP2-54 | —— |
|  | OTU1960 | Module hub | Proteobacteria | Alphaproteobacteria | Elsterales | norank_o__Elsterales | norank_f__norank_o__Elsterales | —— |
|  | OTU2769 | Module hub | Acidobacteriota | Acidobacteriae | Bryobacterales | Bryobacteraceae | Bryobacter | —— |
|  | OTU2642 | Module hub | RCP2-54 | norank_p__RCP2-54 | norank_c__norank_p__RCP2-54 | norank_o__norank_c__norank_p__RCP2-54 | norank_f__norank_o__norank_c__norank_p__RCP2-54 | —— |
|  | OTU2288 | Module hub | Actinobacteriota | Thermoleophilia | Solirubrobacterales | Solirubrobacteraceae | Conexibacter | —— |
|  | OTU1954 | Module hub | Acidobacteriota | Acidobacteriae | Solibacterales | Solibacteraceae | Candidatus_Solibacter | —— |
|  | OTU336 | Connector | Acidobacteriota | Acidobacteriae | Bryobacterales | Bryobacteraceae | Bryobacter | animal_parasites_or_symbionts |
|  | OTU584 | Connector | Proteobacteria | Gammaproteobacteria | Xanthomonadales | Rhodanobacteraceae | Rhodanobacter | chemoheterotrophy |
|  | OTU904 | Connector | Planctomycetota | Planctomycetes | Gemmatales | Gemmataceae | norank_f__Gemmataceae | —— |
|  | OTU1957 | Connector | Proteobacteria | Alphaproteobacteria | Rhizobiales | unclassified_o__Rhizobiales | unclassified_o__Rhizobiales | —— |
|  | OTU67 | Connector | Proteobacteria | Gammaproteobacteria | Burkholderiales | Burkholderiaceae | Cupriavidus | chemoheterotrophy |
|  | OTU63 | Connector | Gemmatimonadota | Gemmatimonadetes | Gemmatimonadales | Gemmatimonadaceae | norank_f__Gemmatimonadaceae | chemoheterotrophy |
|  | OTU1338 | Connector | Acidobacteriota | Holophagae | Subgroup_7 | norank_o__Subgroup_7 | norank_f__norank_o__Subgroup_7 | —— |
|  | OTU3008 | Connector | Acidobacteriota | Vicinamibacteria | Vicinamibacterales | norank_o__Vicinamibacterales | norank_f__norank_o__Vicinamibacterales | —— |
|  | OTU1861 | Connector | Verrucomicrobiota | Verrucomicrobiae | Pedosphaerales | Pedosphaeraceae | norank_f__Pedosphaeraceae | —— |
|  | OTU1819 | Connector | Gemmatimonadota | Gemmatimonadetes | Gemmatimonadales | Gemmatimonadaceae | norank_f__Gemmatimonadaceae | —— |
|  | OTU3444 | Connector | Proteobacteria | Gammaproteobacteria | Burkholderiales | Nitrosomonadaceae | MND1 | —— |
|  | OTU1695 | Connector | Planctomycetota | Planctomycetes | Gemmatales | Gemmataceae | norank_f__Gemmataceae | —— |
|  | OTU374 | Connector | Proteobacteria | Alphaproteobacteria | Elsterales | norank_o__Elsterales | norank_f__norank_o__Elsterales | cellulolysis |
|  | OTU3736 | Connector | Myxococcota | Polyangia | Polyangiales | Polyangiaceae | Pajaroellobacter | —— |
|  | OTU1803 | Connector | Actinobacteriota | Acidimicrobiia | Microtrichales | Ilumatobacteraceae | CL500-29_marine_group | —— |
|  | OTU4220 | Connector | Chloroflexi | Ktedonobacteria | Ktedonobacterales | Ktedonobacteraceae | unclassified_f__Ktedonobacteraceae | —— |
|  | OTU3284 | Connector | Proteobacteria | Alphaproteobacteria | Caulobacterales | Hyphomonadaceae | SWB02 | —— |

**Table S2 The taxonomic composition of keystone in soil fungal community**

| Group | OTU ID | Topological role | Phylum | Class | Order | Family | Genus | Guild |
| --- | --- | --- | --- | --- | --- | --- | --- | --- |
| S1 | OTU1006 | Connector | Ascomycota | Sordariomycetes | Hypocreales | unclassified_o__Hypocreales | unclassified_o__Hypocreales | Undefined Saprotroph |
|  |  |  |  |  |  |  |  |  |
| S2 | OTU2059 | Module hub | Ascomycota | Sordariomycetes | Hypocreales | unclassified_o__Hypocreales | unclassified_o__Hypocreales | Undefined Saprotroph |
|  | OTU1275 | Connector | Ascomycota | Sordariomycetes | Hypocreales | Hypocreaceae | Trichoderma | Undefined Saprotroph |
|  | OTU3841 | Connector | unclassified_k__Fungi | unclassified_k__Fungi | unclassified_k__Fungi | unclassified_k__Fungi | unclassified_k__Fungi | —— |
|  | OTU3483 | Connector | unclassified_k__Fungi | unclassified_k__Fungi | unclassified_k__Fungi | unclassified_k__Fungi | unclassified_k__Fungi | —— |
|  | OTU58 | Connector | Mucoromycota | Umbelopsidomycetes | Umbelopsidales | Umbelopsidaceae | Umbelopsis | Undefined Saprotroph |
|  | OTU2727 | Connector | Basidiomycota | Tremellomycetes | Trichosporonales | Trichosporonaceae | Apiotrichum | Soil Saprotroph |
|  | OTU4498 | Connector | unclassified_k__Fungi | unclassified_k__Fungi | unclassified_k__Fungi | unclassified_k__Fungi | unclassified_k__Fungi | —— |
|  | OTU160 | Connector | Ascomycota | unclassified_p__Ascomycota | unclassified_p__Ascomycota | unclassified_p__Ascomycota | unclassified_p__Ascomycota | —— |
|  |  |  |  |  |  |  |  |  |
| S3 | OTU505 | Module hub | Ascomycota | Sordariomycetes | Xylariales | Xylariaceae | Hypoxylon | Undefined Saprotroph |
|  | OTU2310 | Module hub | unclassified_k__Fungi | unclassified_k__Fungi | unclassified_k__Fungi | unclassified_k__Fungi | unclassified_k__Fungi | —— |
|  | OTU2089 | Module hub | unclassified_k__Fungi | unclassified_k__Fungi | unclassified_k__Fungi | unclassified_k__Fungi | unclassified_k__Fungi | —— |
|  | OTU1568 | Connector | unclassified_k__Fungi | unclassified_k__Fungi | unclassified_k__Fungi | unclassified_k__Fungi | unclassified_k__Fungi | —— |
|  | OTU1500 | Connector | unclassified_k__Fungi | unclassified_k__Fungi | unclassified_k__Fungi | unclassified_k__Fungi | unclassified_k__Fungi | —— |
|  | OTU2199 | Connector | Ascomycota | Sordariomycetes | Hypocreales | Bionectriaceae | unclassified_f__Bionectriaceae | Undefined Saprotroph |
|  | OTU745 | Connector | Ascomycota | Dothideomycetes | Pleosporales | Biatriosporaceae | Biatriospora | Endophyte-Wood Saprotroph-Animal Pathogen |
|  |  |  |  |  |  |  |  |  |
| S4 | OTU125 | Module hub | Ascomycota | unclassified_p__Ascomycota | unclassified_p__Ascomycota | unclassified_p__Ascomycota | unclassified_p__Ascomycota | —— |
|  | OTU1494 | Module hub | unclassified_k__Fungi | unclassified_k__Fungi | unclassified_k__Fungi | unclassified_k__Fungi | unclassified_k__Fungi | —— |
|  | OTU4494 | Connector | unclassified_k__Fungi | unclassified_k__Fungi | unclassified_k__Fungi | unclassified_k__Fungi | unclassified_k__Fungi | —— |
|  | OTU1871 | Connector | Ascomycota | Leotiomycetes | Helotiales | unclassified_o__Helotiales | unclassified_o__Helotiales | —— |
|  |  |  |  |  |  |  |  |  |
| Wet season | OTU1892 | Module hub | Ascomycota | unclassified_p__Ascomycota | unclassified_p__Ascomycota | unclassified_p__Ascomycota | unclassified_p__Ascomycota | —— |
|  | OTU1419 | Module hub | unclassified_k__Fungi | unclassified_k__Fungi | unclassified_k__Fungi | unclassified_k__Fungi | unclassified_k__Fungi | —— |
|  | OTU1505 | Connector | Ascomycota | Sordariomycetes | Sordariales | Chaetomiaceae | unclassified_f__Chaetomiaceae | Animal Pathogen-Dung Saprotroph-Endophyte-Epiphyte-Plant Saprotroph-Wood Saprotroph |
|  | OTU423 | Connector | Ascomycota | Sordariomycetes | Hypocreales | Hypocreaceae | Trichoderma | Undefined Saprotroph |
|  |  |  |  |  |  |  |  |  |
| Dry season | OTU101 | Module hub | Ascomycota | Eurotiomycetes | Chaetothyriales | Herpotrichiellaceae | Cladophialophora | Undefined Saprotroph |
|  | OTU423 | Module hub | Ascomycota | Sordariomycetes | Hypocreales | Hypocreaceae | Trichoderma | Undefined Saprotroph |
|  | OTU354 | Module hub | Ascomycota | Sordariomycetes | Chaetosphaeriales | Chaetosphaeriaceae | unclassified_f__Chaetosphaeriaceae | Plant Saprotroph-Wood Saprotroph |
|  | OTU96 | Module hub | Ascomycota | Sordariomycetes | Chaetosphaeriales | Chaetosphaeriaceae | Chloridium | Ectomycorrhizal |
|  | OTU2385 | Module hub | unclassified_k__Fungi | unclassified_k__Fungi | unclassified_k__Fungi | unclassified_k__Fungi | unclassified_k__Fungi | —— |
|  | OTU1631 | Module hub | Ascomycota | Sordariomycetes | Hypocreales | Clavicipitaceae | Paecilomyces | Undefined Saprotroph |
|  | OTU2766 | Module hub | unclassified_k__Fungi | unclassified_k__Fungi | unclassified_k__Fungi | unclassified_k__Fungi | unclassified_k__Fungi | —— |
|  | OTU2538 | Module hub | Ascomycota | Eurotiomycetes | Chaetothyriales | Herpotrichiellaceae | Phialophora | Animal Pathogen-Endophyte-Ericoid Mycorrhizal-Plant Pathogen-Wood Saprotroph |
|  | OTU4011 | Connector | unclassified_k__Fungi | unclassified_k__Fungi | unclassified_k__Fungi | unclassified_k__Fungi | unclassified_k__Fungi | —— |
|  | OTU462 | Connector | Ascomycota | Sordariomycetes | Hypocreales | Clavicipitaceae | Metarhizium | Animal Pathogen |
|  | OTU2134 | Connector | unclassified_k__Fungi | unclassified_k__Fungi | unclassified_k__Fungi | unclassified_k__Fungi | unclassified_k__Fungi | —— |
|  | OTU817 | Connector | Mortierellomycota | Mortierellomycetes | Mortierellales | Mortierellaceae | Mortierella | Endophyte-Litter Saprotroph-Soil Saprotroph-Undefined Saprotroph |
|  | OTU1514 | Connector | unclassified_k__Fungi | unclassified_k__Fungi | unclassified_k__Fungi | unclassified_k__Fungi | unclassified_k__Fungi | —— |
|  | OTU122 | Connector | Ascomycota | Eurotiomycetes | Eurotiales | Aspergillaceae | Penicillium | Undefined Saprotroph |
|  | OTU4519 | Connector | Ascomycota | Leotiomycetes | Helotiales | unclassified_o__Helotiales | unclassified_o__Helotiales | —— |
|  | OTU20 | Connector | Ascomycota | Sordariomycetes | Hypocreales | Cordycipitaceae | Simplicillium | Animal Pathogen |
|  | OTU2757 | Connector | Ascomycota | Leotiomycetes | Helotiales | unclassified_o__Helotiales | unclassified_o__Helotiales | —— |
|  | OTU2793 | Connector | Ascomycota | unclassified_p__Ascomycota | unclassified_p__Ascomycota | unclassified_p__Ascomycota | unclassified_p__Ascomycota | —— |
|  | OTU4561 | Connector | Mortierellomycota | Mortierellomycetes | Mortierellales | Mortierellaceae | Mortierella | Endophyte-Litter Saprotroph-Soil Saprotroph-Undefined Saprotroph |
|  | OTU4440 | Connector | Ascomycota | Dothideomycetes | Capnodiales | Teratosphaeriaceae | Devriesia | Plant Pathogen |
|  | OTU1399 | Connector | Ascomycota | unclassified_p__Ascomycota | unclassified_p__Ascomycota | unclassified_p__Ascomycota | unclassified_p__Ascomycota | —— |
|  | OTU4104 | Connector | Ascomycota | unclassified_p__Ascomycota | unclassified_p__Ascomycota | unclassified_p__Ascomycota | unclassified_p__Ascomycota | —— |
|  | OTU1820 | Connector | unclassified_k__Fungi | unclassified_k__Fungi | unclassified_k__Fungi | unclassified_k__Fungi | unclassified_k__Fungi | —— |
